# Evidence for an Association between Post-Fledging Dispersal and Microsatellite Multilocus Heterozygosity in a Large Population of Greater Flamingos

**DOI:** 10.1371/journal.pone.0081118

**Published:** 2013-11-22

**Authors:** Mark A. F. Gillingham, Frank Cézilly, Rémi Wattier, Arnaud Béchet

**Affiliations:** 1 Université de Bourgogne, Equipe Ecologie Evolutive, UMR CNRS 6282 Biogéosciences, Dijon, France; 2 Centre de Recherche de la Tour du Valat, Le Sambuc, Arles, France; 3 Leibniz Institute for Zoo and Wildlife Research, dpt. of Evolutionary Genetics, Berlin, Germany; 4 Insitut Universitaire de France, Paris, France; University of Texas, United States of America

## Abstract

Dispersal can be divided into three stages: departure, transience and settlement. Despite the fact that theoretical studies have emphasized the importance of heterozygosity on dispersal strategies, empirical evidence of its effect on different stages of dispersal is lacking. Here, using multi-event capture-mark-recapture models, we show a negative association between microsatellite multilocus heterozygosity (MLH; 10 loci; *n* = 1023) and post-fledging dispersal propensity for greater flamingos, *Phoenicopterus roseus*, born in southern France. We propose that the negative effects of inbreeding depression affects competitive ability and therefore more homozygous individuals are more likely to disperse because they are less able to compete within the highly saturated natal site. Finally, a model with the effect of MLH on propensity of post-fledgling dispersers to disperse to the long-distance sites of Africa was equivalent to the null model, suggesting that MLH had low to no effect on dispersal distance. Variations in individual genetic quality thus result in context-dependent heterogeneity in dispersal strategies at each stage of dispersal. Our results have important implications on fitness since sites visited early in life are known to influence site selection later on in life and future survival.

## Introduction

Dispersal is a crucial life history trait in natural populations, with numerous implications for individual fitness as well as for species' distribution, population dynamics and genetics [1]–[3]. As recently highlighted by Clobert *et al.* [2], dispersal can be separated into three stages: departure, transience and settlement. The extrinsic factors, such as kin competition, resource competition, and environmental conditions, and, intrinsic factors, such as sex, age and juvenile body condition (or early body condition; hereafter referred to as EBC) are known to be some of the factors that influence dispersal strategies, and are likely to act independently on each stage of dispersal [1]–[3]. In most species of birds and mammals, juveniles are more likely to disperse than adults [4]. Although, traditionally, studies have focused on natal dispersal (dispersal to the first breeding site) and breeding dispersal (dispersal between successive breeding sites) as important fitness traits [4], dispersal of juvenile sexually immature individuals (referred to as post-fledging dispersal in birds [5]) may also have important fitness implications. For instance, in the greater flamingo (*Phoenicopterus roseus*), juvenile long-distance dispersers suffer lower survival in the first two years of life than juvenile residents or juvenile intermediate-distance dispersers [6].

The importance of heterozygosity has long been suggested as an important intrinsic factor in dispersal by theoretical studies [7]–[12], but it has been the subject of few empirical studies. Recent studies have used neutral markers, mainly microsatellites, to evaluate the association of multi-locus heterozygosity (MLH) with fitness traits, referred to as heterozygosity-fitness correlation (HFC). HFC has been found across several taxa [13], [14] with potential consequences on dispersal strategies [15]–[18]. Two main hypotheses have been proposed to explain HFC. First, the “general effect” hypothesis suggests that the heterozygosity of neutral markers reflects that of functional markers, each one having a small effect on polygenic traits [13], [14]. This association in heterozygosity is predicted to occur if recurrent inbreeding and/or outbreeding results in a correlation in heterozygosity and/or homozygosity across loci, known as identity equilibrium (ID) [14]. A second hypothesis is the so-called “local effects” hypothesis, whereby observed HFC is generated by a few localized loci due to either “indirect effects” or “direct effects”. HFC generated by “indirect local effects” occurs if the contribution of a few markers, linked to functional non-neutral genes, exceeds that of the sum of all loci. However, theory predicts that HFC due to the indirect effects of linkage of non-functional markers to functional markers implies some degree of inbreeding and/or outbreeding in the population since linkage enhances but does not generate association between markers [14]. In contrast, HFC generated by “direct local effects” occurs if the markers used to estimate heterozygosity have a direct effect on fitness and thus functional overdominance occurs independently of inbreeding [13], [14], [19], [20]. However, when estimating heterozygosity using microsatellite markers, “direct effects” have often been dismissed, based on the assumption that microsatellites are predominantly selectively neutral and non-functional [13], [14] (but see Olano-Marin, Mueller & Kempenaers [21], [22]).

Regardless of the mechanisms involved, MLH has been predicted to influence dispersal in two different ways. First, heterozygosity may be negatively associated with dispersal propensity [18]. Dispersal is often costly, both in terms of energy expenditure and survival. In addition, unfamiliar surrounding areas may be not suitable or less favorable habitats than the natal area [1]–[3]. However, habitat saturation may force less competitive individuals to leave their natal area and disperse [23], [24]. If competitive ability is related to heterozygosity, a negative association between MLH and dispersal can then be expected. Indeed, Shafer *et al.*'s [18] study of mountain goats (*Oreamnos americanus*) in western North America provides some empirical evidence that heterozygosity is negatively associated with dispersal propensity. Second, the negative effects of inbreeding depression, namely the increased expression of recessive deleterious alleles and the loss of heterozygote advantage, could decrease the capacity of dispersing and result in a positive association between heterozygosity and dispersal distance, as found in great tits, *Parus major* [16] and the Siberian flying squirrels, *Petromys volans* [17]. Surprisingly, given the importance of heterozygosity in dispersal theory, other than the few studies mentioned above, we know of no other empirical study which investigated whether dispersal is associated with heterozygosity. Furthermore, although both hypotheses are not mutually exclusive, there is to date no empirical evidence that heterozygosity may have a differential effect on dispersal according to the stage of dispersal. For instance, heterozygosity may be negatively associated with dispersal propensity (i.e. departure) if the natal area is saturated and competition is high, but positively associated with dispersal distance (i.e. transience and settlement) if inbreeding depression negatively impacts dispersal ability.

In this study we take advantage of a large dataset of greater flamingos born in the highly saturated and competitive site of the Camargue (southern France), to evaluate the relative influences of microsatellite multilocus heterozygosity (MLH) on both post-fledging dispersal propensity and the propensity for post-fledging dispersers to disperse long distances, while controlling for survival and resighting probabilities, using multi-event capture-mark-recapture models [25].

## Methods

### Collection of samples

**Figure 1:**
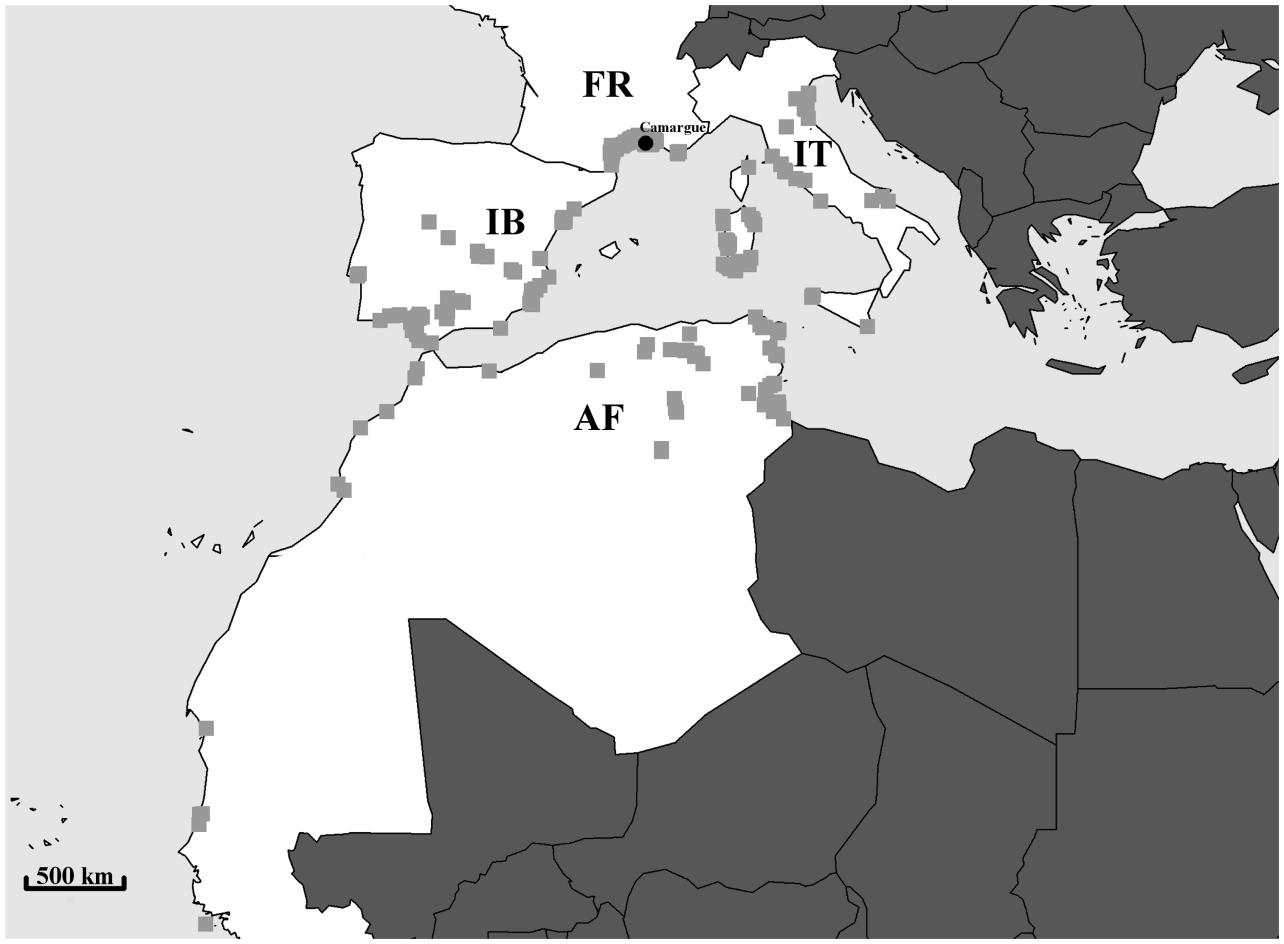
Map showing the natal site in Camargue, southern France and resightings locations of greater flamingos from summer 1995 to winter 2010 in the Mediterranean and west Africa which are represented by the grey squares. Also shown are the geographical regions used in this study: southern France (FR); Italy (IT); Iberian Peninsula (IB); and, north and west Africa (AF).

Greater flamingos inhabit brackish wetlands of the Mediterranean basin where they breed at a limited number of sites (<15) [26]. Between 1995 and 1998 in July/August, ∼800 (800 to 954) chicks were caught by herding the crèche into a corral at the only breeding site in southern France, the Fangassier's lagoon (Figure 1; 43°25′N, 4°37′E; Salin-de-Giraud, Camargue). On average, capture was carried out 106 days after the start of egg laying, just before the oldest chicks fledged. Thus a random sample of chicks, which included early- and late-hatching birds, was captured (approximate age range 35–77 days) [26]. During ringing operations, we measured tarsus length to the nearest 1 mm and body weight to the nearest 50 g using a 5-kg Pesola spring balance (see Johnson & Cézilly [26] for details) and we collected blood samples for DNA extraction and sexing (for details see Gillingham *et al.* [27]). Chicks were also marked individually with PVC plastic rings engraved with a four-digit code which can be read at up to 300 m. Greater flamingos lay only one egg per season, systematically switch mates between consecutive seasons and show strong age-assortative mating [26], [28], [29], excluding any full-sibling relationships in the present study.

### Ethics Statement

Ringing and sample collection of greater flamingo chicks were authorised through the personal permit (number 405) of Alan Johnson and Arnaud Béchet delivered by the Centre de Recherche sur la Biologie des Populations d'Oiseaux (CRBPO, Muséum national d'histoire naturelle, France). The study protocol was reviewed and approved by the CRBPO.

### Study area and field methods

Resightings of ringed flamingos were achieved from winter 1995 to winter 2010 through a network of professional and amateur ornithologists across the Mediterranean and west Africa. We only retained resightings during the winter months of November-January and during the summer months of March-August to avoid the problem of including observations recorded during migratory stopovers. If more than one observation was made during these time intervals, we retained the observation that was the furthest away in terms of distance from the previous observation.

Since we were interested in post-fledging dispersal, and not natal or breeding dispersal, the breeding status of the birds in summer was not considered. In the first fall following fledging, there is high dispersal (above 50%) to the first wintering site (November-January) in the greater flamingo [6], [30]. Most flamingos do not generally reach sexual maturity until the age of 5 [31] and have never been observed to breed below the age of 3 [32]. Although much lower, there is also a propensity to move in the latter stages in life of greater flamingo, with movements between winter (November-January) and summer sites (March-August) and reciprocally between summer and winter sites. Therefore evaluating the effects of EBC and MLH on the movements of birds from fledging to the first winter is equivalent to investigating their effects on post-fledging dispersal but not on natal dispersal in this species.

Although survival and dispersal may typically be biased by unnoticed long-distance dispersers outside the range of the study area, this bias is minimized in our case since the study area was large and included almost all major sites of the Mediterranean population except for Turkey (Figure 1), namely: southern France (FR; range distance from natal site 0–200 km), Italy (IT; range distance from natal site 495–1373 km), the Iberian peninsula (IB; range distance from natal site 456–1603 km), and north and west Africa (AF; range distance from natal site 797–4222 km). Furthermore during the time period of our study, only 0.88% of resightings for the studied cohorts were outside the study area. Therefore although survival and dispersal probabilities must be considered as local to the study area they are likely to be representative of true survival and dispersal.

While several sites of Italy and the Iberian Peninsula are further away from the natal site than north and west Africa, frequent stopover sites in Italy and the Iberian Peninsula appears to facilitate dispersal to these geographical regions [30], [33], [34]. Importantly post-fledgling dispersal to north and west Africa involves crossing the Mediterranean sea during which they seldomly rest and which has been shown to be associated with lower survival in a previous study [6]. For simplicity, we therefore refer to post-fledgling dispersal to Italy and the Iberian Peninsula as “intermediate-distance dispersal” and post-fledgling dispersal to north and west Africa as “long-distance dispersal”.

### Microsatellite genotyping

In addition to the 670 individuals genotyped by Gillingham *et al.* [27], 353 more individuals (a total of 1023) were genotyped for ten microsatellite loci (PrD3, PrD4, PrD5, PrD9, PrA110, PrA113, PrC109, PrD108, PrD121 and PrD126) developed for the greater flamingo (see [27] or details of microsatellite genotyping). For each cohort, the program MICRO-CHECKER [35] was used to test for genotyping errors. Identity disequilibrium (ID) of the 10 microsatellite loci used in this study, was estimated from parameter *g_2_*, using the RMES software [36]. Multilocus heterozygosity (MLH) was measured as the sum of the single loci heterozygosity (SLH), with missing values replaced by the mean at that locus, divided by the number of loci [14]. This measure of MLH enables the comparison of models with all individual SLH to models with MLH and to investigate whether a potential MLH effect on a life-history trait is driven by local effects [14]. Although other measures of heterozygosity exist which are highly collinear with MLH [see supporting information of Gillingham *et al.* [27] for correlation between MLH and other indices of heterozygosity], only MLH is presented here following recommendations from Chapman *et al.* [13].

### Early body condition

The scaled mass index was used to calculate EBC [37], [38], which controls for the effects of body size on both the independent and dependent variables. Chick tarsus length was used as a body size indicator, which significantly correlates with chicks' mass [27], [30], [39], [40]. When calculating the scaled mass index, the slope of the standard major axis (SMA) of the natural log-transformed mass against the natural log-transformed tarsus length for all cohorts and both sexes combined was used as the scaling exponent using the R function SMATR. This was legitimate because of a lack of difference in slopes (*p*>0.30) when fitting a SMA regression of the natural log-transformed mass against the natural log-transformed tarsus within each cohort and sex [38]. Therefore, despite morphological differences between sexes and cohorts, the same morphogenetic pattern for males and females between each cohort was assumed.

### Multi-event capture-resighting analyses

#### (i)Goodness-of-fit

We tested the goodness-of-fit of a general multistate model assuming full-time variation of resighting, survival and transition parameters (the Jolly Movement model) [41] using U-CARE 2.2.2 [42], [43]. The test was significant (Table S1 in Appendix S1), indicating a lack of fit due to changes in resighting, survival and transition parameters with age and other cohort-dependent factors. Indeed, as found by Sanz-Aguilar *et al* [6], the same test was no longer significant after taking into account full cohort-dependent factors (Table S2 in Appendix S1). However because a full-cohort-dependent model with other individual covariates such as EBC and MLH would be over parameterized, we decided to enter the cohort effect for the first age class of survival and movement probabilities and to use the inflation factor based on the lack of fit of the model without the cohort effect. This enabled us to only keep the strongest effects of cohort in the early life stages while remaining on the safe side by scaling down model deviances to account for any remaining lack of fit. The inflation factor was calculated as the ratio of the goodness-of-fit statistic to its degrees of freedom [44] and was ĉ = 1.61 (Table S1 in Appendix S1).

#### (ii)Multi-event modeling structure

We evaluated the effect of cohort, age, sex, season (winter/summer), EBC and MLH on the probabilities of survival, movement and destination of dispersers using E-SURGE 1.7.1 [45]. We encoded observations in multi-event encounter histories as in Sanz-Aguilar *et al.* [6]. In multi-event modeling the events are what is observed in the field and may be distinguished from the underlying biological states. Here, the events were: ‘0’ – bird not resighted, ‘1’ – bird marked/resighted in France, ‘2’ – bird resighted in Italy, ‘3’ – bird resighted in the Iberian Peninsula, ‘4’ – bird resighted in north and west Africa. The underlying biological states considered were: ‘FR’ – bird spent season in France, ‘IT’ – bird spent season in Italy, ‘IB’ – bird spent season in the Iberian Peninsula, ‘AF’ – bird spent season in north and west Africa, and ‘D’ – dead bird.

Three kinds of parameters are estimated in multi-event models: the initial state probabilities, the probabilities of transition between states, and the probabilities of the events conditional on the underlying states. The initial state probabilities were not used since every individual in our data set was ringed as a chick in France. Transition probabilities were decomposed to distinguish 3 steps: survival; movement probability irrespective of destination; and, the probability of reaching a given destination for dispersers. This decomposition enabled us to evaluate different effects on each step. The event probabilities 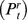 for all models correspond to the time-specific probabilities of resighting in the different areas; which was estimated using the following matrix: 
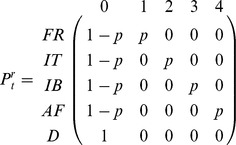



Previous studies in greater flamingos have demonstrated that survival and dispersal differ between juveniles and adults. Therefore to avoid over-parameterizing our models, we limited the decomposition of age classes to two groups and evaluated different age class composition. In addition to the cohort effect for the 1^st^ age class of survival and movement probability, we also included a seasonal effect (winter/summer) for movement probability and destination, since movements vary across seasons. We then evaluated the fit of models that differ in the age structures considered, cohort effects, the effect of season and sex on the three transition steps (Table S3 in Appendix S2). Models without the covariates EBC and MLH were run using repeated random initial values in order to ensure convergence of models on the global minima (*n* = 5) [46].

We used the best-supported model with the lowest number of parameters as a null model to evaluate EBC and MLH as predictors of survival or dispersal. EBC and MLH were mean-centered before being included in E-SURGE as covariates. Models with the covariates, EBC and MLH were run using the initial values of the null model as a starting point in order to ensure convergence of models on the global minima without having to repeat the time consuming models 5 times (using the “last model” option in E-SURGE) [46]. The relative importance of each predictor variable was estimated by summing the AIC weight (ΣAIC*ω*) in which that variable appears across supported models (models with a cumulative *ω* of 0.95) [47], [48]. A summed Akaike weight value tends towards 1 if a particular predictor appears in all of the supported models. Conversely, a summed Akaike weight value tends towards 0 if a particular predictor appears only in models with low support. In addition to the summed Akaike weight, we considered that a parameter had support in predicting the data only if 95% confidence intervals of the estimate did not overlap zero. Parameter estimates were calculated using model averaging whereby regression coefficients are averaged across models with a cumulative *ω* of 0.95 and weighted by the Akaike weight of the model in which the coefficients appear, which are more robust when several models have similar support [47], [49].

#### (iii) Modeling the effect of EBC and MLH on survival

The matrix for the first transition step, survival 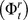 at the site of departure *r* and for the time-interval *t* to *t*+1, was: 
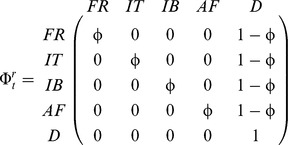



This transition step enabled us to assess the effect of EBC and MLH on the first age class of the survival transition step (<2 year-old, i.e. from ringing to the second winter; referred to hereafter as juvenile survival). In addition, this transition step enabled us to evaluate the effect of EBC and MLH on the survival of juveniles that had dispersed, dispersed to intermediate-distance sites and dispersed to long distance sites.

#### (iv) Modeling the effect of EBC and MLH on dispersal propensity

The second transition step describes the probability of movement from a given area (France: *dispFR*; Italy: *dispIT*; Iberian Peninsula: *dispIB*; Africa: *dispAF*) irrespective of the destination, and its complement, the fidelity to an area (France: *fidFR*; Italy: *fidIT*; Iberian Peninsula: *fidIB*; Africa: *fidAF*) 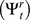 for birds alive at site r at time t and alive at site *s* at *t*+1. The matrix for this second transition step was: 
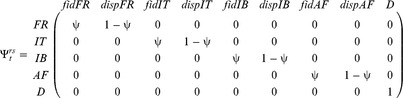



This transition step enabled us to evaluate the effect of MLH and EBC on the first age class of movement probability (i.e. from ringing to the first wintering site; hereafter referred to as post-fledging dispersal). Since the best model retained an effect of MLH (see results), we repeated the best model with MLH replaced by all individual SLH to evaluate whether the association was better explained by “local” effects [14].

#### (v) Modeling the effect of EBC and MLH on propensity of long-distance dispersal among post-fledging dispersers

The third and last transition step corresponds to the destination of dispersing individuals individuals 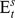:
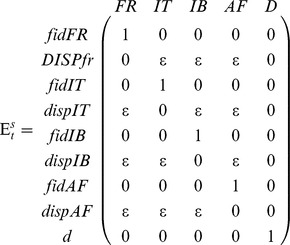



This decomposition of transition steps allowed us to perform most of the models. However, in models assessing the association of EBC and MLH on propensity for post-fledging dispersers to disperse long distances, we concatenated columns 2 and 3 of *dispFR* for the first age class only (i.e. only for the destination post-fledging dispersers). This enabled us to evaluate the propensity among post-fledging dispersers to disperse to “the long-distance sites of Africa”, and its complement, “the intermediate-distance sites of Italy and the Iberian Peninsula” according to MLH and EBC. Once again since the best model retained an effect of MLH (see results), we repeated the best model with MLH replaced by all individual SLH to evaluate whether the association was better explained by “local” effects [14].

## Results

### Identity disequilibrium

We found no significant evidence of ID (*g_2_* = −0.00076, SD = 0.002, *p* = 0.377) and heterozygosity-heterozigosity correlations gave equivalent results (Table S4 in Appendix S3).

### Null multi-event model

Model selection allowed us to consider the following model structure as the null model to evaluate the effect of individual covariates (for full details of null model selection see Appendix S2):

- Juvenile survival was cohort-dependent but adult survival was not (Figure S1 in Appendix S2).
- Movement probabilities differed between two age classes. Post-fledging dispersal was independent of sex, season (winter or summer) and cohort while adult movements were season- and site-dependent (Figure S2 in Appendix S2).
- Destinations differed between the two age classes. Post-fledging dispersal destination was independent of sex, season and cohort while adult movement destination was site-dependent but independent of season (Figure S3 in Appendix S2).
- Resighting probabilities were time and site-specific.

### Association between MLH and EBC, and survival

We evaluated an association between MLH and EBC, and the survival of: juveniles (models 25 and 24 respectively); juvenile dispersers (models 20 and 18 respectively); long-distance juvenile dispersers (models 21 and 22 respectively); and, intermediate-distance juvenile dispersers (models 23 and 19 respectively). Since the null model had the smallest QAICc, there was low support of an association between MLH or EBC and juvenile survival (Table 1; ΣAIC*ω*<0.16 for MLH and EBC).

**Table 1:** Model selection for models evaluating an association between the life history trait of juvenile survival and the individual covariates of microsatellite multilocus heterozygosity (MLH) and early body condition (EBC).

Model	Description	*np*	Deviance	QAICc	ΔQAICc	*ω*
**13**	**Null model: Φ_(a_Juvenile___.cohort)+a_Adult__, Ψ_a_Fledgling___+(a_Adult___.season)_, E_a_Fledgling___+a_Adult__**	**148**	**25823.42**	**16326.40**	**0.00**	**0.241**
18	Association between EBC and survival of juvenile dispersers (survival of juveniles who have dispersed to Italy, the Iberian Peninsula and Africa): Φ_(a_Fledgling__ _.cohort)+a_JuvenileFR__ _+(a_JuvenileIT,IB,AF__ _.EBC)+a_Adult__, Ψ_a_Fledgling__ _+(a_Adult__ _.season)_, E_a_Fledgling__ _+a_Adult__	151	25814.54	16327.25	0.86	0.157
19	Association between EBC and survival of juvenile intermediate-distance dispersers (survival of juveniles who have dispersed to Italy and the Iberian Peninsula): Φ_(a_Fledgling__ _.cohort)+a_JuvenileFR,AF__ _+(a_JuvenileIT,IB__ _.EBC)+a_Adult__, Ψ_a_Fledgling__ _+(a_Adult__ _.season)_, E_a_Fledgling__ _+a_Adult__	151	25814.76	16327.39	0.99	0.147
20	Association between MLH and survival of juvenile dispersers (survival of juveniles who have dispersed to Italy, the Iberian Peninsula and Africa): Φ_(a_Fledgling__ _.cohort)+a_JuvenileFR__ _+(a_Juvenile IT,IB,AF__ _.MLH)+a_Adult__, Ψ_a_Fledgling__ _+(a_Adult__ _.season)_, E_a_Fledgling__ _+a_Adult__	151	25815.06	16327.57	1.18	0.134
21	Association between MLH and survival of juvenile long-distance dispersers (survival of juveniles who have dispersed to Africa): Φ_(a_Fledgling__ _.cohort)+a_JuvenileFR,IT,IB__ _+(a_Juvenile AF__ _.MLH)+a_Adult__, Ψ_a_Fledgling__ _+(a_Adult__ _.season)_, E_a_Fledgling__ _+a_Adult__	151	25815.52	16327.86	1.47	0.116
22	Association between EBC and survival of juvenile long-distance dispersers (survival of juveniles who have dispersed to Africa): Φ_(a_Fledgling__ _.cohort)+a_JuvenileFR,IT,IB__ _+(a_JuvenileAF__ _.EBC)+a_Adult__, Ψ_a_Fledgling__ _+(a_Adult__ _.season)_, E_a_Fledgling__ _+a_Adult__	151	25815.74	16328.00	1.60	0.108
23	Association between MLH and survival of juvenile intermediate-distance dispersers (survival of juveniles who have dispersed to Italy and the Iberian Peninsula): Φ_(a_Fledgling__ _.cohort)+a_JuvenileFR,AF__ _+(a_JuvenileIT,IB__ _.MLH)+a_Adult__, Ψ_a_Fledgling__ _+(a_Adult__ _.season)_, E_a_Fledgling__ _+a_Adult__	151	25816.83	16328.68	2.28	0.077
24	Association between EBC and juvenile survival: Φ_(a_Juvenile__ _.cohort.EBC)+a_Adult__, Ψ_a_Fledgling__ _+(a_Adult__ _.season)_, E_a_Fledgling__ _+a_Adult__	152	25819.21	16332.27	5.88	0.013
25	Association between MLH and juvenile survival: Φ_(a_Juvenile__ _.cohort.MLH)+a_Adult__, Ψ_a_Fledgling__ _+(a_Adult__ _.season)_, E_a_Fledgling__ _+a_Adult__	152	25821.58	16333.74	7.35	0.006

The best model (lowest QAICc) is in bold. Number of parameters (*np*), the delta QAICc (ΔQAICc) calculated from the null model 13 and model weight (*ω*) are given. Age class is symbolized by the letter “a”: juvenile encompasses the first two years of life; adult survival encompasses >2 years and older; fledgling encompasses the first winter and adult movement all successive seasons following the first winter. Geographical location is symbolized as “FR” (France), “IT” (Italy), “IB” (Iberian Peninsula) and “AF” (Africa).

### Association between MLH and EBC, and post-fledging dispersal propensity

**Figure 2:**
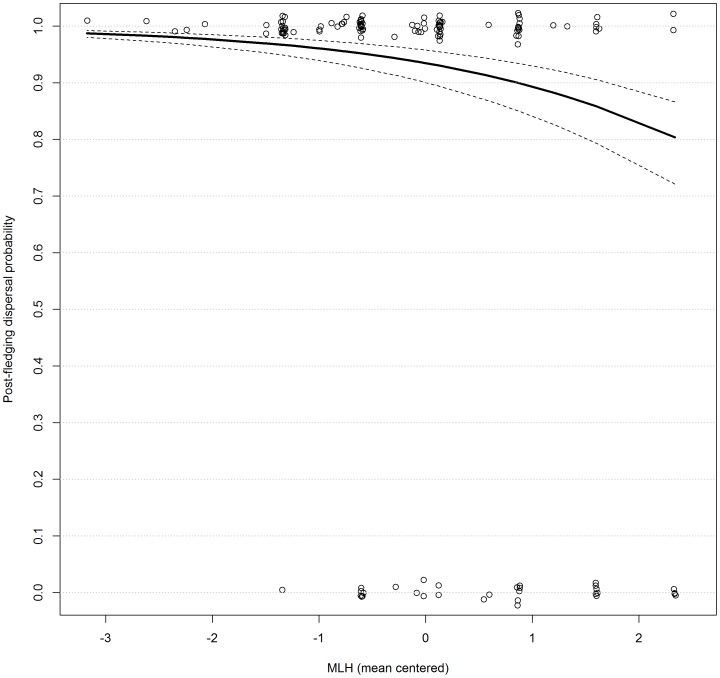
Probability of post-fledging dispersal according to microsatellite multilocus heterozygosity (MLH) for greater flamingos born in Camargue, southern France. 95% confidence intervals correspond to the broken lines. Model averaged estimates were extracted from models 26 and 27 (see Table 2). Circles represent the observations of whether birds that have been resighted in their first wintering site (although all estimates from models 26 and 27 are based on all 1023 individuals) dispersed (1) or not (0) for a given MLH value for a given MLH value. Overlapping points have been jittered to give a better indication of sample size for each MLH value.

Model selection revealed support for an association between MLH and post-fledging dispersal propensity (Table 2; model 26; ΔQAICc = −3.82; model 27; ΔQAICc = −2.03; ΣAIC*ω* = 0.86), with more homozygous individuals more likely to disperse than heterozygotes (Figure 2: slope  = −0.54, 95%CI = −0.99, −0.08). In contrast, there was low support for an association between post-fledging dispersal propensity and EBC (Table 2; model 28; ΔQAICc = 1.02; ΣAIC*ω* = 0.25) and adding EBC to MLH as an additive predictor of post-fledgling dispersal propensity did not improve QAICc (Table 2; model 27 versus model 26). When entering all SLH as individual predictors of post-fledging dispersal propensity, they were not retained as better predictors than MLH (Table 2; model 29 versus model 26; ΣAIC*ω* = 0).

**Table 2:** Model selection for models evaluating an association between the life history trait of post-fledging dispersal propensity and the individual covariates of microsatellite multilocus heterozygosity (MLH) and early body condition (EBC).

Model	Description	*np*	Deviance	QAICc	ΔQAICc	*ω*
**26**	Association between MLH and post-fledging dispersal probability: Φ_(a_Juvenile__ _.cohort)+a_Adult__, Ψ_(a_Fledgling__ _.MLH) +(a_Adult__ _.season)_, E_a_Fledgling__ _+a_Adult__	149	25813.84	16322.57	−3.82	0.608
27	Association between EBC and MLH; and post-fledging dispersal probability: Φ_(a_Juvenile__ _.cohort)+a_Adult__, Ψ_(a_Fledgling__ _.(EBC+MLH)) +(a_Adult__ _.season)_, E_a_Fledgling__ _+a_Adult__	150	25813.31	16324.37	−2.03	0.248
13	Null model: Φ_(a_Juvenile__ _.cohort)+a_Adult__, Ψ_a_Fledgling__ _+(a_Adult__ _.season)_, E_a_Fledgling__ _+a_Adult__	148	25823.42	16326.40	0.00	0.090
28	Association between EBC and post-fledging dispersal probability: Φ_(a_Juvenile__ _.cohort)+a_Adult__, Ψ_(a_Fledgling__ _.EBC) +(a_Adult__ _.season)_, E_a_Fledgling__ _+a_Adult__	149	25821.65	16327.42	1.03	0.054
29	Association between each of the 10 SLH used to calculate MLH and post-fledging dispersal probability: Φ_(a_Juvenile__ _.cohort)+a_Adult__, Ψ_(a_Fledgling__ _.(SLH1+ SLH2+ SLH3+ SLH4+ SLH5+ SLH6+ SLH7+ SLH8+ SLH9+ SLH10)) +(a_Adult__ _.season)_, E_a_Fledgling__ _+a_Adult__	158	25803.96	16335.57	9.17	0.001

The best model (lowest QAICc) is in bold. Number of parameters (*np*), the delta QAICc (ΔQAICc) calculated from the null model 13 and model weight (*ω*) are given. Age class is symbolized by the letter “a”: juvenile encompasses the first two years of life; adult survival encompasses >2 years and older; fledgling encompasses the first winter and adult movement all successive seasons following the first winter.

### Association between MLH and EBC, and propensity of post-fledging dispersers to disperse long distances

Model 30, which modeled the effect of MLH on propensity to disperse to the long-distance sites of Africa among post-fledging dispersers, was only marginally better supported in terms of AIC than the null model (Table 3; model 30; ΔQAICc = −0.32). The summed AIC weight (ΣAIC*ω* = 0.55) suggested low support for MLH to be positively associated with long-distance dispersal to the sites of Africa and 95% confidence intervals overlapped 0 (slope  = 0.24, 95%CI = −0.06, 0.54). There was low support for EBC to predict post-fledging dispersal to the long-distance sites of Africa (Table 3; model 33; ΔQAICc = 1.00; ΣAIC*ω* = 0.22). When entering all SLH as individual predictors of propensity of post-fledging dispersers to disperse long distances, they were not retained as better predictors than MLH (Table 3; model 34 versus model 31; ΣAIC*ω* = 0).

**Table 3:** Model selection for models evaluating an association between the life history trait of propensity of post-fledging dispersers to disperse long-distances (the intermediate-distance sites of Italy and Iberian Peninsula or the long-distance sites of Africa) and the individual covariates of microsatellite multilocus heterozygosity (MLH) and early body condition (EBC).

Model	Description	*np*	Deviance	QAICc	ΔQAICc	*ω*
**30**	**Association between MLH and probability of post-fledging dispersers to disperse to the long-distances sites (Africa): Φ_(a_Juvenile___.cohort)+a_Adult__, Ψ_a_Fledgling___+(a_Adult___.season)_, E_(a_Fledgling___.MLH)+a_Adult__**	**148**	**25826.69**	**16328.42**	**−0.31**	**0.329**
31	Null model: Φ_(a_Juvenile__ _.cohort)+a_Adult__, Ψ_a_Fledgling__ _+(a_Adult__ _.season)_, E__Fledgling__ _+a_Adult__	147	25830.61	16328.74	0.00	0.281
32	Association between EBC and MLH; and probability of post-fledging dispersers to disperse to the long-distances sites of Africa: Φ_(a_Juvenile__ _.cohort)+a_Adult__, Ψ_a_Fledgling__ _+(a_Adult__ _.season)_, E_(a_Fledgling__ _.(MLH+EBC))+a_Adult__	149	25824.58	16329.24	0.50	0.219
33	Association between EBC and probability of post-fledging dispersers to disperse to the long-distances sites of Africa: Φ_(a_Juvenile__ _.cohort)+a_Adult__, Ψ_a_Fledgling__ _+(a_Adult__ _.season)_, E_(a_Fledgling__ _.EBC)+a_Adult__	148	25828.81	16329.74	1.00	0.170
34	Association between each of the 10 SLH used to calculate MLH and probability of post-fledging dispersers to disperse to the long-distances sites of Africa: Φ_(a_Juvenile__ _.cohort)+a_Adult__, Ψ_a_Fledgling__ _+(a_Adult__ _.season)_, E_(a_Fledgling__ _.(SLH1+ SLH2+ SLH3+ SLH4+ SLH5+ SLH6+ SLH7+ SLH8+ SLH9+ SLH10))+a_Adult__	157	25820.30	16343.58	14.84	<0.001

The best model (lowest QAICc) is in bold. Number of parameters (*np*), the delta QAICc (ΔQAICc) calculated from the null model 31 and model weight (*ω*) are given. Age class is symbolized by the letter “a”: juvenile encompasses the first two years of life; adult survival encompasses >2 years and older; fledgling encompasses the first winter and adult movement all successive seasons following the first winter.

## Discussion

We found support for a negative association between post-fledging dispersal propensity and MLH in the greater flamingo. In addition, we found that a model with the effect of MLH on propensity of post-fledgling dispersers to disperse to the long-distance sites of Africa was equivalent to the null model. We found low support for EBC to predict both post-fledging dispersal propensity and distance. Finally, we found low support for EBC- or MLH-dependent juvenile survival.

We found support for post-fledging dispersal propensity to be associated with MLH. In the absence of a direct effect of microsatellite MLH on fitness, a correlation between MLH and fitness traits is only predicted to occur if there is a correlation in heterozygosity across loci, known as identity disequilibrium (ID) [14]. Although we found no evidence of ID in our population, slight inbreeding is more easily detected through its effects on phenotype than through its effects on heterozygosity at a few marker loci [14]. Indeed, most studies reporting significant heterozygosity-fitness correlation (HFC) fail to find significant ID [14] (but see Olano-Marin, Mueller & Kempenaers [21] for a rare example of significant ID). How well microsatellite MLH predicts inbreeding depression remains a contentious issue especially when using a relatively small number of microsatellite markers such as in this study (10) [13], [14]. However, a recent study on a large (*n* = 1192) captive population of zebra finches (*Taeniopygia guttata*) found that 11 microsatellite markers and 1359 SNP markers produced equally strong heterozygosity-fitness correlation (HFC) on 11 phenotypic traits [50]. Furthermore both markers produced stronger HFCs than the pedigree based *F* [50]. Although ID was not tested for in this study, the population was known *a priori* to be moderately inbred. Forstmeier *et al.* [50] suggest that since a loss of hereterozysity is the root of inbreeding depression, measures of heterozygosity, even those based on a small panel of microsatelittes, are likely to be more informative in measuring the realized effect of inbreeding depression on fitness traits than previously anticipated.

In random mating scenarios, the “general effect” hypothesis predicts that a significant correlation between fitness traits and MLH at neutral markers may occur due to a recent bottleneck and/or admixture between different populations [14]. However a bottleneck is unlikely to explain our results since the average time since expansion from the last bottleneck was estimated to be 696,421 years (90% CI: 526 316–1 131 579 yr) in the greater flamingo [51]. Alternatively, if there is frequent immigration and admixture from genetically distinct populations outbreeding depression may occur resulting in a breakup of coadapted gene complexes or favorable epistatic interactions [52]. Outbreeding depression would then generate a positive relationship between MLH and a fitness trait. A negative association between post-fledging dispersal propensity and MLH could thus be explained by a positive association between MLH and survival, whereby there is differential survival between homozygotes and heterozygotes during dispersal. This is particularly relevant for post-fledgling dispersal to the long-distance sites of Africa which involves the hazardous crossing of the Mediterranean Sea and has previously been demonstrated to be costly in terms of survival [6]. However, we found no evidence for MLH-dependent survival, regardless of whether they dispersed long-distances or not. Indeed we found no evidence that survival of juveniles, juvenile dispersers, juvenile intermediate-distance dispersers or juvenile long-distance dispersers was associated with MLH.

An alternative hypothesis is that a few localized loci that are closely associated with functional loci (“indirect local effect”) and that may have a strong effect on fitness or have a direct effect on fitness (“direct local effect”) generate a correlation between MLH and fitness. We could not identify whether loci used in this study were presumably non-functional as recently achieved in a population of blue tits, *Cynistes caeruleus*, by Olano-Marin, Mueller & Kempenaers [21], [22]. However, we found no evidence that models with all individual SLH better explain the data than a model with MLH, suggesting that our results were not driven by a few localized loci.

Finally, a correlation between MLH and fitness traits can also occur if there is frequent consanguineous mating in the population [14]. Yet, in the absence of pedigree information on this greater flamingo population, the level of consanguineous mating remains unknown in our population. Since the greater flamingos form a single large interbreeding population in the Mediterranean [51] and given the fact that they switch mates between consecutive breeding seasons [28], frequent consanguineous mating may, at first, seem unlikely. However in the greater flamingo there seems to be no sex-specific natal dispersal [53], which is thought to be an effective mechanism to avoid inbreeding in birds [3], [16]. Furthermore there are a limited number of Mediterranean breeding sites for the greater flamingo [26], which varies from year to year depending on favorable climatic conditions and local water levels [26], [39], [40]. Therefore consanguineous mating might be more frequent than previously anticipated in this population of greater flamingos and might explain the correlation between MLH and post-fledgling dispersal.

Regardless of the mechanisms involved, the results in both this study and Shafer *et al.*'s [18] provide correlative evidence of a negative association between MLH and dispersal propensity. Several factors suggest that the decision of fledglings to disperse may be determined by competition in the natal site. The Camargue is a high quality site, with the highest breeding success in the Mediterranean, but it is also known to be under the most intense competition due to saturation, with young inexperienced birds queuing to breed [29], [32], [39], [53], [54]. Furthermore, recent evidence suggests that flamingos breeding for the first time in Camargue are more likely, than more experienced breeders, to either be forced to skip breeding or to move to another presumably less competitive site the next year [54]. Further suggesting that competitive-ability is age related is that younger inexperienced individuals are less likely to settle in high quality nesting sites [55]. Therefore, since more than 50% of fledglings (92% for the cohorts used in this study; Figure S2 in Appendix S2) disperse to other sites in the Mediterranean for their first winter, high levels of post-fledging dispersal propensity is likely to be driven by age related competitive ability. Although in our study MLH was not associated with EBC (Table S5 and Table S6 in Appendix S4; and Figure S4 in Appendix S4) or survival, more homozygous individuals are associated with a decrease in fitness and competitive ability in many species [13], [14]. We therefore suggest that the negative effects of inbreeding depression on competitive ability of fledglings increases the probability of homozygous fledglings to disperse from the natal site as a result of high competition.

The effect of MLH on post-fledgling dispersal propensity may have important implications for later life history traits, since greater flamingos tend to select the same wintering sites they have visited during their first and second year of life [6], [30], [34]. Furthermore, as predicted by the arrival-time hypothesis [56], birds who have settled earlier and who have greater experience of their future breeding site are likely to have a higher reproductive success because they are more likely to acquire a high quality mate and access high quality nesting space, an important factor of breeding success in this species [26], [55]. Since site fidelity is much higher after the first winter (between 72–92% in our study; Figure S2 in Appendix S2), birds that have remained in France during their first winter are likely to have a competitive advantage compared to birds that have dispersed when queuing for nesting in their high-quality natal site. Given that the most heterozygous individuals were ∼20% more likely to remain in their natal site during their first winter than the most homozygous individuals (Figure 2), this may represent a considerable fitness advantage for heterozygote individuals. Further studies could test this by investigating whether heterozygotes are more likely than homozygotes to be first-time breeders and to queue for less time to breed in their high quality natal site in the Camargue.

The decision to disperse is likely to be costly in greater flamingos. Barbraud, Johnson & Bertault [30] found that EBC was associated with post-fledging dispersal propensity and post-fledging dispersal to the long-distance site of Africa, suggesting that dispersal is costly in terms of energy expenditure. In the present study, we found low support for post-fledging dispersal probability to be associated with EBC, which may be explained by the additional cohort used in Barbraud, Johnson & Bertault's [30] study. Alternatively, Barbraud, Johnson & Bertault [30] did not control for both spatial and temporal variation in resighting probabilities, as achieved in this study, which may have biased their estimates. However stronger evidence of the cost of dispersal comes from Sanz-Aguilar *et al.*'s [6] study that shows that juvenile greater flamingos that dispersed to the long-distance sites of Africa had lower survival. Despite this cost, north and west African sites were the most likely destinations of post-fledging dispersers (73%; Figure S3 in Appendix S2) confirming previous results for the same cohorts [6], [30]. We found that a model with the effect of MLH on propensity to disperse to the long-distance sites of Africa was equivalent to the null model. MLH may therefore have no effect on post-fledgling dispersal distance. Alternatively, we may have lacked the statistical power to detect the weaker positive effect of MLH on post-fledging dispersal distance (approximately half as strong as the effect on post-fledgling dispersal propensity) among fledglings that have chosen to disperse. The latter suggests that homozygotes post-fledging dispersers may tend to select the intermediate-distance sites of Italy or the Iberian Peninsula and heterozygotes the long-distance sites of Africa. One possibility, then, is that the negative effect of inbreeding depression also negatively affects dispersal ability during transience and that homozygotes were less able to disperse to the longer-distance sites of Africa than heterozygote dispersers, which would be consistent with what has been found in great tits, *Parus major* [16] and Siberian flying squirrels, *Petromys volans* [17]. The fact that there was either no effect of MLH on dispersal distance or a weaker effect of MLH on dispersal distance than on dispersal propensity can be explained by the more complex extrinsic factors that may affect dispersal transience which are outside the scope of this paper. Indeed, the ability of greater flamingos to disperse to the long-distance sites of Africa have been shown to be explained by weather conditions and in particular favourable dominant winds [34]. In addition, long-distance dispersers have been shown to stop to rest in small wetlands prior to reaching their destination [33], which may vary in availability and levels of competition according to the dispersal route chosen (e.g. through Italy or Iberian Peninsula) and local weather conditions.

In summary, we propose that during the departure stage of dispersal, more homozygous individuals were more likely to disperse in our study population because they were less able to compete within the high quality but highly saturated sites of southern France. In contrast during the transience stage of dispersal, we suggest that the lack of MLH effect on dispersal distance among dispersers may be due to the more complex extrinsic factors influencing dispersal during transience and settlement. Our results are therefore in accordance with theoretical predictions that variation in individual quality will result in context-dependent heterogeneity in dispersal strategies at each stage of dispersal [2]. This is likely to result in complex patterns in the association between dispersal and MLH, and intrinsic factors in general, which may vary both within and between species depending on the extrinsic factors of the natal and alternative settlement sites.

## Supporting Information

Appendix S1 **Goodness-of-fit tests of capture-mark-recapture models for the MLH.** Table S1: Results of the different components of goodness-of-fit tests for the general model. Table S2: Results of the global goodness-of-fit tests for the general model by cohort.
(DOCX)

Appendix S2 **Model selection of age classes, cohort effects, seasonal effects and sex effects.** Table S3: Model selection for the optimal null model. Figure S1: Estimates of survival (95% confidence intervals) of flamingos according to model 13. Figure S2: Estimates of post-fledging dispersal and subsequent movement probabilities (95% confidence intervals) of flamingos according to model 13. Figure S3: Probabilities (95% confidence intervals) of moving to different destinations by Greater flamingos at post-fledging dispersal and in subsequent years according to model 13.
(DOCX)

Appendix S3 **Heterozygosity–heterozygosity correlations using the R package ‘Rhh’.** Table S4: Heterozygosity–heterozygosity correlations.
(DOCX)

Appendix S4 **Repeating Gillingham** ***et al.*** **'s** [**27**] **analysis testing for an association between EBC and MLH with a larger sample size.** Table S5: Models of early body condition of greater flamingos. Table S6: Model averaged parameter estimates of models predicting early body condition of greater flamingos. Figure S4: Adjusted mean early body condition (±SEM) of greater flamingo chicks according to cohort and sex (using model 1 in Table S5).
(DOCX)
