# Supplementary material for: Evidence for an Association between Post-Fledging Dispersal and Microsatellite Multilocus Heterozygosity in a Large Population of Greater Flamingos
Source: PLoS One. 2013 Nov 22;8(11):e81118. doi: 10.1371/journal.pone.0081118 (PMC3838344; doi:10.1371/journal.pone.0081118)
Supplement: Appendix S1 — Goodness-of-fit tests of capture-mark-recapture models for the MLH. Table S1: Results of the different components of goodness-of-fit tests for the general model. Table S2: Results of the global goodness-of-fit tests for the general model by cohort. (DOCX) [file pone.0081118.s001.docx]

**Appendix S1: Goodness-of-fit tests of capture-mark-recapture models for the MLH**

Table S1 : Results of the different components of goodness-of-fit tests for the general model. Significant tests are indicated by the ‘*’ symbol.

| Test | χ2 | df | ĉ |
| --- | --- | --- | --- |
| WBWA* | 378.933 | 141 |  |
| 3G.SR* | 13.725 | 3 |  |
| 3G.SM | 343.586 | 378 |  |
| M.ITEC* | 267.136 | 114 |  |
| M.LTEC* | 229.637 | 129 |  |
| Global Test* | 1233.017 | 765 | 1.611787 |

Table S2 : Results of the global goodness-of-fit tests for the general model by cohort. No test was statistically significant.

| Test | χ2 | df | ĉ |
| --- | --- | --- | --- |
| 1995 | 218.334 | 257 |  |
| 1996 | 120.479 | 158 |  |
| 1997 | 437.023 | 389 |  |
| 1998 | 338.944 | 297 |  |
| Global | 1114.78 | 1101 | 1.012516 |
