# Supplementary material for: Evidence for an Association between Post-Fledging Dispersal and Microsatellite Multilocus Heterozygosity in a Large Population of Greater Flamingos
Source: PLoS One. 2013 Nov 22;8(11):e81118. doi: 10.1371/journal.pone.0081118 (PMC3838344; doi:10.1371/journal.pone.0081118)
Supplement: Appendix S2 — Model selection of age classes, cohort effects, seasonal effects and sex effects. Table S3: Model selection for the optimal null model. Figure S1: Estimates of survival (95% confidence intervals) of flamingos according to model 13. Figure S2: Estimates of post-fledging dispersal and subsequent movement probabilities (95% confidence intervals) of flamingos according to model 13. Figure S3: Probabilities (95% confidence intervals) of moving to different destinations by Greater flamingos at post-fledging dispersal and in subsequent years according to model 13. (DOCX) [file pone.0081118.s002.docx]

**Appendix S2: Model selection of age classes, cohort effects, seasonal effects and sex effects**

The optimal age classes were to combine the first 4 seasons after fledging for survival (first two years of age, hereafter referred to as juveniles) and to separate the first season (post-fledging dispersal) from subsequent seasons (adult movement) for movement probability and movement destination probability (model 4; Table S3). A model without a cohort effect for post-fledging dispersal (model 10; Table S3) was better supported than a model with a cohort effect for post-fledging dispersal (model 4; ΔQAICc=4.58; Table S3). In addition a model without a seasonal effect (winter/summer) for adult movement destination (model 8; Table S3) was better supported than a model with this effect (model 4; ΔQAICc=2.96; Table S3). Therefore a model without the cohort effect for juvenile survival and seasonal effect for movement destination (model 13; Table S3) had a lower QAICc than a model with both of these effects (model 4; ΔQAICc=7.51; Table S3). A model which tested for sex dependent post-fledging dispersal (models 14; Table S3) was equivalent to a model without a sex effect (ΔQAICc=-0.88).

Sanz-Aguilar *et al.* (2012) found that juvenile long-distance dispersers (AF) had lower survival than non-dispersers (FR) and intermediate-distance dispersers (IB and IT). In our study we found low support for differential juvenile survival between long-distance dispersers (AF) and the rest (FR, IT and IB; model 16; Table S3), for differential juvenile survival between dispersers (IT, IB and AF) and non-dispersers (FR; model 15; Table S3), and for differential juvenile survival between intermediate-distance dispersers (IB and IT) and the rest (FR and AF; model 17; Table S3). Indeed models 15, 16 and 17 gave equivalent QAICc values (ΔQAICc=0.04; ΔQAICc=-0.10 and ΔQAICc=0.20 respectively) to model 13 which assumes equal juvenile survival between geographical sites. However Sanz-Aguilar *et al.*’s (2012) study uses a considerably larger sample size (*n*= 22 671) and larger number of cohorts (*n*= 33) than this study, which we believe is the most likely explanation for differences in results. We selected the best supported model with the lowest number of parameters to avoid over parameterization to test the association of parameters, EBC and MLH on survival and post-fledging dispersal, which was model 13 (Appendix S5, Supporting information). However models 15, 16 and 17 were respectively used as a base model to set initial values when evaluating the effect of EBC and MLH on the survival of juveniles: that dispersed (models 20 and 18), that dispersed to long distance sites (models 21 and 22), and that dispersed to intermediate-distance sites (models 23 and 19).

According to model 13, differential juvenile survival between cohorts was predominantly driven by lower survival of the 1998 cohort (Figure S1; 88.36%, 95CI: 85.60%, 90.64%) compared to the 1995 (Figure S1; 93.66%, 95CI: 90.41%, 95.85%), 1996 (Figure S1; 93.29%, 95CI: 88.92%, 96.02%) and 1997 (Figure S1; 91.97%, 95CI: 89.63%, 93.81%) cohorts. Adult survival (>2 years) was higher at 98.55% (Figure S1; 95CI: 98.12%, 98.89%). Consistent with previous studies post-fledging dispersal was high at 92.15% (Figure S2; 95CI: 86.72%, 95.47%) with much lower adult movement varying between 8% and 26%. The most frequent destination of fledglings was the African sites at 73% (Figure S3; 95CI: 64.91%, 80.32%) with 17.51% (Figure S3; 95CI: 11.73%, 25.32%) dispersing to sites in the Iberian Peninsula and 9.17% (Figure S3; 95CI: 5.78%, 14.25%) dispersing to sites in Italy.

Table S3 : Model selection for the optimal null model for age classes, cohort effects, seasonal effects and sex effects for each transitional step in order to test the association between the life history traits of survival, post-fledging dispersal and post-fledging distance and the individual parameters of microsatellite multi locus heterozygosity (MLH) and early body condition (EBC). The optimal model used as the null model (lowest number of *np* between models of equivalent QAICc) is in bold. Number of parameters (*np*), the delta QAICc (ΔQAICc) calculated from model 13 and model weight (*ω*) are given.

| Model | Description | *np* | Dev | QAICc | ΔQAICc | *ω* |
| --- | --- | --- | --- | --- | --- | --- |
| 1 | **Φ_(a(1).cohort)+a(2:32)_, Ψ_(a(1).cohort)+(a(2:32).season)_, ε_(a(1))+(a(2:32).season)_**  Two age classes for survival probability: 1^st^ summer (post-fledgling) and following seasons  Two age classes for movement probability and destination probability: 1^st^ summer (post-fledgling) and following seasons  Cohort effect for the 1st age classes of survival probability and destination probability  Season effect for the 2^nd^ age classes of movement probability and destination probability | 159 | 25838.00 | 16358.82 | 32.42 | 0.000 |
| 2 | **Φ_(a(1:2).cohort)+a(3:32)_, Ψ_(a(1).cohort)+(a(2:32).season)_, ε_(a(1))+(a(2:32).season)_**  Two age classes for survival : from 1^st^ summer to 1^st^ winter and following seasons  Two age classes for movement probability and destination probability: 1^st^ summer (post-fledgling) and following seasons  Cohort effect for the 1st age classes of survival probability and destination probability  Season effect for the 2^nd^ age classes of movement probability and destination probability | 159 | 25816.04 | 16345.19 | 18.79 | 0.000 |
| 3 | **Φ_(a(1:3).cohort)+a(4:32)_, Ψ_(a(1).cohort)+(a(2:32).season)_, ε_(a(1))+(a(2:32).season)_**  Two age classes for survival : from 1^st^ summer to 2^nd^ summer and following seasons  Two age classes for movement probability and destination probability: 1^st^ summer and following seasons  Cohort effect for the 1st age classes of survival probability and destination probability  Season effect for the 2^nd^ age classes of movement probability and destination probability | 159 | 25800.27 | 16335.41 | 9.01 | 0.002 |
| 4 | **Φ_(a(1:4).cohort)+a(5:32)_, Ψ_(a(1).cohort)+(a(2:32).season)_, ε_(a(1))+(a(2:32).season)_**  Two age classes for survival : from 1^st^ summer to 2^nd^ winter and following seasons  Two age classes for movement probability and destination probability: 1^st^ winter and following seasons  Cohort effect for the 1st age classes of survival probability and destination probability  Season effect for the 2^nd^ age classes of movement probability and destination probability | 159 | 25797.85 | 16333.91 | 7.51 | 0.004 |
| 5 | **Φ_(a(1:5).cohort)+a(6:32)_, Ψ_(a(1).cohort)+(a(2:32).season)_, ε_(a(1))+(a(2:32).season)_**  Two age classes for survival : from 1^st^ summer to 3^rd^ summer and following seasons  Two age classes for movement probability and destination probability: 1^st^ winter and following seasons  Cohort effect for the 1st age classes of survival probability and destination probability  Season effect for the 2^nd^ age classes of movement probability and destination probability | 159 | 25801.10 | 16335.92 | 9.52 | 0.001 |
| 6 | **Φ_(a(1:6).cohort)+a(7:32)_, Ψ_(a(1).cohort)+(a(2:32).season)_, ε_(a(1))+(a(2:32).season)_**  Two age classes for survival : from 1^st^ summer to 4^th^ summer and following seasons  Two age classes for movement probability and destination probability: 1^st^ winter and following seasons  Cohort effect for the 1st age classes of survival probability and destination probability  Season effect for the 2^nd^ age classes of movement probability and destination probability | 159 | 25811.02 | 16342.08 | 15.68 | 0.000 |
| 7 | **Φ_(a(1:4).cohort)+a(5:32)_, Ψ_(a(1:2).cohort)+(a(3:32).season)_, ε_(a(1))+(a(2:32).season)_**  Two age classes for survival : from 1^st^ summer to 4^th^ summer and following seasons  Cohort effect for the 1st age classes of survival probability and destination probability  Two age classes for movement probability and destination probability: 1^st^ winter to 1^st^ summer and following seasons  Season effect for the 2^nd^ age classes of movement probability and destination probability | 177 | 25774.17 | 16357.68 | 31.28 | 0.000 |
| 8 | **Φ_(a(1:4).cohort)+a(5:32)_, Ψ_(a(1).cohort)+ a(2:32.season)_, ε_(a(1))+(a(2:32))_**  Two age classes for survival : from 1^st^ summer to 2^nd^ winter and following seasons  Two age classes for movement probability and destination probability: 1^st^ winter and following seasons  Cohort effect for the 1st age classes of survival probability and destination probability  Season effect for the 2^nd^ age classes of movement probability | 151 | 25820.50 | 16330.95 | 4.56 | 0.017 |
| 9 | **Φ_(a(1:4).cohort)+a(5:32)_, Ψ_(a(1))+(a(2:32))_, ε_(a(1))+(a(2:32).season)_**  Two age classes for survival : from 1^st^ summer to 2^nd^ winter and following seasons  Two age classes for movement probability and destination probability: 1^st^ winter and following seasons  Cohort effect for the 1st age class of survival probability  Season effect for the 2^nd^ age classes of destination probability | 155 | 25838.11 | 16350.38 | 23.98 | 0.000 |
| 10 | **Φ_(a(1:4).cohort)+a(5:32)_, Ψ_(a(1))+(a(2:32).season)_, ε_(a(1))+(a(2:32).season)_**  Two age classes for survival : from 1^st^ summer to 2^nd^ winter and following seasons  Two age classes for movement probability and destination probability: 1^st^ winter and following seasons  Cohort effect for the 1st age class of survival probability  Season effect for the 2^nd^ age classes of movement probability and destination probability | 156 | 25800.76 | 16329.33 | 2.93 | 0.039 |
| 11 | **Φ_(a(1:4))+a(5:32)_, Ψ_(a(1).cohort)+(a(2:32).season)_, ε_(a(1))+(a(2:32).season)_**  Two age classes for survival : from 1^st^ summer to 2^nd^ winter and following seasons  Two age classes for movement probability and destination probability: 1^st^ winter and following seasons  Cohort effect for the 1st age class of movement probability  Season effect for the 2^nd^ age classes of movement probability and destination probability | 156 | 25814.57 | 16337.90 | 11.50 | 0.001 |
| 12 | **Φ_(a(1:4).cohort)+(a(5:32).season)_,Ψ_(a(1).cohort)+(a(2:32).season)_, ε_(a(1))+(a(2:32).season)_**  Two age classes for survival : from 1^st^ summer to 2^nd^ winter and following seasons  Two age classes for movement probability and destination probability: 1^st^ winter and following seasons  Cohort effect for the 1st age classes of survival probability and destination probability  Season effect for the 2^nd^ age classes of survival probability, movement probability and destination probability | 160 | 25797.64 | 16335.90 | 9.51 | 0.001 |
| 13 | **Φ_(a(1:4).cohort)+a(5:32)_, Ψ_a(1)+(a(2:32).season)_, ε_(a(1))+(a(2:32))_**  **Two age classes for survival : from 1^st^ summer to 2^nd^ winter and following seasons**  **Two age classes for movement probability and destination probability: 1^st^ winter and following seasons**  **Cohort effect for the 1st age classes of survival probability**  **Season effect for the 2^nd^ age class of movement probability** | **148** | **25823.42** | **16326.40** | **0.00** | **0.170** |
| 14 | **Φ_(a(1:4).cohort)+a(5:32)_, Ψ_(a(1).sex)+(a(2:32).season)_, ε_(a(1))+(a(2:32))_**  Sex effect for the 1^st^ age class of movement probability | 149 | 25818.58 | 16325.52 | -0.88 | 0.264 |
| 15 | **Φ_(a(1).cohort)+a(2:4)FR+a(2:4)IT,IB,AF+ a(5:32)_, Ψ**_a(1)_**_+(a(2:_**_32_**_).season)_, ε_(a(1))+(a(2:_**_32_**_))_**  Differential mortality between juveniles that have dispersed (in Italy, Iberian Peninsula and Africa) and juveniles that are in the natal site (France) | 150 | 25816.64 | 16326.43 | 0.04 | 0.167 |
| 16 | **Φ_(a(1).cohort)+ a(2:4)FR,IT,IB+a(2:4)AF+ a(5:32)_, Ψ**_a(1)_**_+(a(2:_**_32_**_).season)_, ε_(a(1))+(a(2:_**_32_**_))_**  Differential mortality between juveniles that have dispersed to long-distance sites (Africa) and juveniles that have not dispersed to long-distance sites (France, Italy and Iberian Peninsula) | 150 | 25816.42 | 16326.30 | -0.10 | 0.179 |
| 17 | **Φ_(a(1).cohort)+ a(2:4)FR,AF+a(2:4)IT,IB+ a(5:32)_, Ψ**_a(1)_**_+(a(2:_**_32_**_).season)_, ε_(a(1))+(a(2:_**_32_**_))_**  Differential mortality between juveniles that have dispersed to intermediate-distance sites (Italy and Iberian Peninsula) and juveniles that have not dispersed to intermediate-distance sites (France and Africa) | 150 | 25816.90 | 16326.59 | 0.20 | 0.154 |

Age class is symbolized by the letter “a”, numbers in brackets represent season number (1 being the first winter and 32 the last winter) and the “:” symbolizes the concatenation of seasons into a single group (e.g. a(1:4) represents the age class: from first winter to 4^th^ summer, i.e. the first two years of life). Geographical location is symbolized as “FR” (France), “IT” (Italy), “IB” (Iberian Peninsula) and “AF” (Africa).

**Figure S1 :** Estimates of survival (95% confidence intervals) of flamingos according to model 13.

**Figure S2 :** Estimates of post-fledging dispersal and subsequent movement probabilities (95% confidence intervals) of flamingos according to model 13.

**Figure S3 :** Probabilities (95% confidence intervals) of moving to different destinations by Greater flamingos at post-fledging dispersal and in subsequent years according to model 13.

**References**

Sanz-Aguilar, A., Béchet, A., Germain, C., Johnson, A.R. & Pradel, R. (2012) To leave or not to leave: survival trade-offs between different migratory strategies in the greater flamingo. *Journal of Animal Ecology*, **81**, 1171–1182.
