# Supplementary material for: Evidence for an Association between Post-Fledging Dispersal and Microsatellite Multilocus Heterozygosity in a Large Population of Greater Flamingos
Source: PLoS One. 2013 Nov 22;8(11):e81118. doi: 10.1371/journal.pone.0081118 (PMC3838344; doi:10.1371/journal.pone.0081118)
Supplement: Appendix S3 — Heterozygosity–heterozygosity correlations using the R package ‘Rhh’. Table S4: Heterozygosity–heterozygosity correlations. (DOCX) [file pone.0081118.s003.docx]

Appendix S3: Heterozygosity–heterozygosity correlations using the R package ‘Rhh’

Table S4 : Heterozygosity–heterozygosity correlations (Balloux *et al.* 2004) for 1023 individuals using 10 microsatellite loci for standardized heterozygosity (SH; Coltman *et al.* 1999); internal relatedness (IR; Amos *et al.* 2001); homozygosity by loci (HL; Aparicio *et al.* 2006) the using the R package ‘Rhh’ (Alho *et al.* 2010) with 1000 randomizations.

| Heterozygosity estimate | Mean *r* | 95% Quantile |
| --- | --- | --- |
| SH | -0.030 | -0.068, 0.012 |
| IR | -0.030 | -0.069, 0.007 |
| HL | -0.031 | -0.068, 0.006 |

**References**

Alho, J.S., Välimäki, K. & Merilä, J. (2010) Rhh: an R extension for estimating multilocus heterozygosity and heterozygosity–heterozygosity correlation. *Molecular Ecology Resources*, **10**, 720–722.

Amos, W., Wilmer, J.W., Fullard, K., Burg, T.M., Croxall, J.P., Bloch, D. & Coulson, T. (2001) The influence of parental relatedness on reproductive success. *Proceedings of the Royal Society of London. Series B: Biological Sciences*, **268**, 2021–2027.

Aparicio, J.M., Ortego, J. & Cordero, P.J. (2006) What should we weigh to estimate heterozygosity, alleles or loci? *Molecular Ecology*, **15**, 4659–4665.

Balloux, F., Amos, W. & Coulson, T. (2004) Does heterozygosity estimate inbreeding in real populations? *Molecular Ecology*, **13**, 3021–3031.

Coltman, D.W., Pilkington, J.G., Smith, J.A. & Pemberton, J.M. (1999) Parasite-Mediated Selection against Inbred Soay Sheep in a Free-Living, Island Population. *Evolution*, **53**, 1259.
