# Supplementary material for: Evidence for an Association between Post-Fledging Dispersal and Microsatellite Multilocus Heterozygosity in a Large Population of Greater Flamingos
Source: PLoS One. 2013 Nov 22;8(11):e81118. doi: 10.1371/journal.pone.0081118 (PMC3838344; doi:10.1371/journal.pone.0081118)
Supplement: Appendix S4 — Repeating Gillingham et al. 's [27] analysis testing for an association between EBC and MLH with a larger sample size. Table S5: Models of early body condition of greater flamingos. Table S6: Model averaged parameter estimates of models predicting early body condition of greater flamingos. Figure S4: Adjusted mean early body condition (±SEM) of greater flamingo chicks according to cohort and sex (using model 1 in Table S5). (DOCX) [file pone.0081118.s004.docx]

**Appendix S4: Repeating Gillingham *et al.*’s (2012) analysis testing for an association between EBC and MLH with a larger sample size**

We repeated Gillingham *et al.*’s (2012) analysis but with the additional individuals genotyped for this study using the software package R-2.15.3 (http://www.R-project.org). Namely we modeled the response variable EBC according to MLH, cohort (1995, 1996, 1997 and 1998), sex (female or male) and the two-way interaction between sex and cohort, using a linear model with a Gaussian distribution. All possible candidate models were constructed. Model selection relied on information-theoretic criteria and multi-model inference approach (Burnham & Anderson 2002) which is recommended when dealing with observational data (Johnson & Omland 2004). Akaike’s Information Criterion adjusted for small samples sizes (AICc) was used for model interpretation. AICc weights (*ω*) were used to assess the relative strength of support for models (Burnham & Anderson 2002; Johnson & Omland 2004). Only the 10 top ranked models based on AICc are presented. Parameter estimates were calculated using model averaging whereby regression coefficients are averaged across models with a cumulative *ω* of 0.95, which are more robust when several models have similar support (Burnham & Anderson 2002; Johnson & Omland 2004). The relative importance of each predictor variable was estimated by summing the AIC weight (ΣAIC*ω*) in which that variable appears across supported models (models with a cumulative *ω* of 0.95) (Burnham & Anderson 2002; Symonds & Moussalli 2011). A summed Akaike weight value tends towards 1 if a particular predictor appears in all of the supported models. Conversely, a summed Akaike weight value tends towards 0 if a particular predictor appears only in models with low support (Burnham & Anderson 2002; Symonds & Moussalli 2011). A ΣAIC*ω* value is a relative measure, its interpretation is strongly dependent on the data and the parameters used, and its value is always > 0 even if there is no association between the parameter and the response variable (Burnham & Anderson 2002, p. 345-347; Galipaud *et al.* submitted). We therefore calculated a baseline ΣAIC*ω* for each parameter based on randomization methods (10000 permutations; Burnham & Anderson 2002, p. 345-347; Galipaud *et al.* submitted). This baseline ΣAIC*ω* is the estimated ΣAIC*ω* if the parameter has no association with the response variable EBC and enables direct comparison with the observed ΣAIC*ω* in order to weight the evidence of an association between the parameter and the response variable (Burnham & Anderson 2002, p. 345-347; Galipaud *et al.* submitted).

Two models were retained as equivalent (Δ AICc < 2; Table S5), both of which retained an effect of sex, cohort and the interaction between sex and cohort. There was strong support for an association between EBC and sex (ΣAIC*ω* = 1; baseline ΣAIC*ω* = 0.39; Table S6; Figure S4), cohort (ΣAIC*ω* = 1; baseline ΣAIC*ω* = 0.24; Table S6; Figure S4) and the interaction between sex and cohort (ΣAIC*ω* = 1; baseline ΣAIC*ω* = 0.04; Table S6; Figure S4). Although MLH was retained in one of the two parsimonious models, the ΣAIC*ω* suggests there was no evidence of an association between EBC and MLH (ΣAIC*ω* = 0.37; baseline ΣAIC*ω* = 0.37; Table S5) and 95% confidence intervals of the slope largely overlapped zero (Table S6).

**Table S5**: Models of early body condition of greater flamingos with number of parameters (*k*), log-likelihood (LL), AICc of the models, change in AICc compared to the best-ranked model (Δ AICc), Akaike model weights (*ω*) and cumulative Akaike weight (Cum. *ω*) given. The full model included sex, cohort, microsatellite MLH, and the two-ways interactions between sex and cohort.

| Model rank | Model | *K* | LL | AICc | Δ AICc | *ω* | Cum. *ω* | *R*^2^ |
| --- | --- | --- | --- | --- | --- | --- | --- | --- |
| 1 | Cohort+Sex+Cohort*Sex | 9 | -7031.35 | 14080.9 | 0 | 0.563 | 0.563 | 0.136 |
| 2 | Cohort+Sex+Cohort*Sex +MLH | 10 | -7030.84 | 14081.9 | 1.02 | 0.338 | 0.901 | 0.137 |
| 3 | Cohort+Sex | 6 | -7036.65 | 14085.4 | 4.49 | 0.06 | 0.961 | 0.127 |
| 4 | Cohort+Sex+MLH | 7 | -7036.05 | 14086.2 | 5.32 | 0.039 | 1 | 0.128 |
| 5 | Cohort | 5 | -7043.28 | 14096.6 | 15.74 | 0 | 1 | 0.115 |
| 6 | Cohort+MLH | 6 | -7042.67 | 14097.4 | 16.53 | 0 | 1 | 0.116 |
| 7 | Sex +MLH | 4 | -7099.44 | 14206.9 | 126.04 | 0 | 1 | 0.013 |
| 8 | Sex | 3 | -7100.55 | 14207.1 | 126.24 | 0 | 1 | 0.011 |
| 9 | MLH | 3 | -7104.82 | 14215.7 | 134.78 | 0 | 1 | 0.002 |
| 10 | Intercept | 2 | -7105.96 | 14215.9 | 135.05 | 0 | 1 | 0.000 |

Table S6 : Model averaged parameter estimates of models predicting early body condition of greater flamingos. See table S5 for descriptions of models. The summed AIC weight of each parameter (ΣAIC*ω*) and baseline summed AIC weight (baseline ΣAIC*ω*), calculated as the estimated ΣAIC*ω* if parameter has no association with early body condition, are also given.

|  | Model-averaged Estimate | Adjusted S.E. | 95 % C.I. | ΣAIC*ω* | Baseline ΣAIC*ω* |
| --- | --- | --- | --- | --- | --- |
| Intercept (1995, Females) | 2126.73 | 37.66 | 2052.91, 2200.54 |  |  |
| Cohort |  |  |  | 1 | 0.24 |
| 1996 | 207.13 | 39.00 | 130.69, 283.57 |  |  |
| 1997 | 225.82 | 28.73 | 169.51, 282.13 |  |  |
| 1998 | 181.84 | 28.21 | 126.56, 237.12 |  |  |
| Sex |  |  |  | 1 | 0.39 |
| Males | -61.49 | 35.89 | -131.84, 8.86 |  |  |
| Cohort×Sex: |  |  |  | 1 | 0.04 |
| 1996 (Males) | -110.99 | 57.61 | -223.9, 1.91 |  |  |
| 1997 (Males) | 52.46 | 43.42 | -32.64, 137.56 |  |  |
| 1998 (Males) | -1.00 | 43.94 | -87.12, 85.12 |  |  |
| MLH | -54.38 | 54.19 | -160.59, 51.83 | 0.37 | 0.37 |

Figure S4 : Adjusted mean early body condition (±SEM) of greater flamingo chicks according to cohort and sex (using model 1 in Table S5). Sample sizes are indicated within brackets.

**References**

Alho JS, Välimäki K, Merilä J (2010) Rhh: an R extension for estimating multilocus heterozygosity and heterozygosity–heterozygosity correlation. *Molecular Ecology Resources*, **10**, 720–722.

Amos W, Wilmer JW, Fullard K *et al.* (2001) The influence of parental relatedness on reproductive success. *Proceedings of the Royal Society of London. Series B: Biological Sciences*, **268**, 2021–2027.

Aparicio JM, Ortego J, Cordero PJ (2006) What should we weigh to estimate heterozygosity, alleles or loci? *Molecular Ecology*, **15**, 4659–4665.

Balloux F, Amos W, Coulson T (2004) Does heterozygosity estimate inbreeding in real populations? *Molecular Ecology*, **13**, 3021–3031.

Burnham K, Anderson D (2002) *Model Selection and Multi-Model Inference - A Practical Information-Theoretic Approach*. Springer, New York.

Coltman DW, Pilkington JG, Smith JA, Pemberton JM (1999) Parasite-Mediated Selection against Inbred Soay Sheep in a Free-Living, Island Population. *Evolution*, **53**, 1259.

Galipaud M, Gillingham MAF, Morgan D, Dechaume-Moncharmont F-X (submitted) Caution in the use of Akaike sums of weights for interpretation of parameter importance in model averaging.

Gillingham MAF, Béchet A, Geraci J *et al.* (2012) Genetic polymorphism in dopamine receptor D4 is associated with early body condition in a large population of greater flamingos, Phoenicopterus roseus. *Molecular Ecology*, **21**, 4024–4037.

Johnson JB, Omland KS (2004) Model selection in ecology and evolution. *Trends in Ecology & Evolution*, **19**, 101–108.

Sanz-Aguilar A, Béchet A, Germain C, Johnson AR, Pradel R (2012) To leave or not to leave: survival trade-offs between different migratory strategies in the greater flamingo. *Journal of Animal Ecology*, **81**, 1171–1182.

Symonds MRE, Moussalli A (2011) A brief guide to model selection, multimodel inference and model averaging in behavioural ecology using Akaike’s information criterion. *Behavioral Ecology and Sociobiology*, **65**, 13–21.
